# Water Availability Is the Main Climate Driver of Neotropical Tree Growth

**DOI:** 10.1371/journal.pone.0034074

**Published:** 2012-04-10

**Authors:** Fabien Wagner, Vivien Rossi, Clément Stahl, Damien Bonal, Bruno Hérault

**Affiliations:** 1 Université des Antilles et de la Guyane, UMR ‘Ecologie des Forêts de Guyane’ BP 709, Kourou, France; 2 CIRAD, UMR ‘Ecologie des Forêts de Guyane’, Kourou, France; 3 INRA, UMR ‘Ecologie des Forêts de Guyane’, Kourou, France; 4 INRA, UMR ‘Ecologie et Ecophysiologie Forestière’, Champenoux, France; Ohio State University, United States of America

## Abstract

• Climate models for the coming century predict rainfall reduction in the Amazonian region, including change in water availability for tropical rainforests. Here, we test the extent to which climate variables related to water regime, temperature and irradiance shape the growth trajectories of neotropical trees. • We developed a diameter growth model explicitly designed to work with asynchronous climate and growth data. Growth trajectories of 205 individual trees from 54 neotropical species censused every 2 months over a 4-year period were used to rank 9 climate variables and find the best predictive model. • About 9% of the individual variation in tree growth was imputable to the seasonal variation of climate. Relative extractable water was the main predictor and alone explained more than 60% of the climate effect on tree growth, i.e. 5.4% of the individual variation in tree growth. Furthermore, the global annual tree growth was more dependent on the diameter increment at the onset of the rain season than on the duration of dry season. • The best predictive model included 3 climate variables: relative extractable water, minimum temperature and irradiance. The root mean squared error of prediction (0.035 *mm.d* ^–1^) was slightly above the mean value of the growth (0.026 *mm.d* ^–1^). • Amongst climate variables, we highlight the predominant role of water availability in determining seasonal variation in tree growth of neotropical forest trees and the need to include these relationships in forest simulators to test, in silico, the impact of different climate scenarios on the future dynamics of the rainforest.

## Introduction

Tropical forests are being threatened at an unprecedented scale by global change. The Amazonian region has already experienced severe droughts recently, such as in 1998 and 2005. Temperatures across Amazonia are currently increasing [1] and are expected to continue to increase with a concomitant decrease in precipitation over the next decades [2], [3]. For instance, the HadCM3 model under updated emissions scenarios predicts severe drying events over Amazonia for the XXI*^st^* century [4], [5]. Climate changes in the tropics have become an increasing concern for their potential impacts on the global carbon cycle. Indeed, tropical forests represent a major reservoir of terrestrial carbon, accounting for half of the estimated 558 Pg of carbon stored in vegetation [6] with 86 Pg for the Amazon basin alone [7]. Improving our knowledge on the climate drivers of forest dynamic will enhance our ability to assess the impact of climate change on carbon cycle [8]. Most current studies performed in tropical rain forests have highlighted three major climate drivers of forest dynamics: soil water content, solar irradiance and air temperature.

Rain or lack of rain is often implicitly viewed as the main driver of forest dynamics [9], as annual NPP generally positively correlates with annual amount of precipitation [10] and that rainfall seasonality plays a key role in the forest response to climate variability [11], table 1. When rainfall is less than evapotranspiration, soil moisture is gradually depleted, increasing tensions in the xylem sap that can eventually trigger stomatal closure and other physiological responses [12]. A lack of water availability or rain could limit tree growth. The relation between the amount of rainfall and water availability for trees is not straightforward and is determined by various soil and plant characteristics (permanent wilting point, field capacity, root distribution). Consequently, water stresses are increasingly estimated using Soil Water Balance Models [13], among which are now available some models explicitly designed for tropical forests [14].

**Table 1:** Expected tree growth response to climate variables.

variable	predicted effect^a^	references	process^a^
*REW*	+	[31]	26.6cmphotosynthesis, xylem tension, stomatal closure, leaf flush
			
*rainfall*	+	[9], [10], [12], [19], [66]–[68]	26.6cmphotosynthesis, xylem tension, stomatal closure, leaf flush
	–	[68], [69]	
*T mean*	–	[18], [52], [70]–[72]	photosynthesis kinetic, stomatal closure
*T min*	–	[19], [54], [73]	photosynthesis kinetic, stomatal closure
	no	[74], [75]	
*T max*	–	[18], [71]	photosynthesis kinetic, stomatal closure
	+	[76]	
	no	[74], [75]	
*VPD*	no	[22], [23]	stomatal closure, transpiration
*irradiance*	+	[15]–[17], [28], [60], [73], [77]	photosynthesis, phenology
	–	[17]	
	no	[23], [73], [78]	
U*	+	[24]	photosynthesis, transpiration

a : expected growth response to the climate variable: (+) trees are expected to grow faster with high values of the climate variable, (–) trees are expected to grow slower with high values of the climate variable.

b : biological processes involve in the tree growth response to a given climate variation.

Irradiance is obviously directly linked to the plant photosynthetic ability through the Photosynthetic Photon Flux Density (PPFD), in turn driving carbon uptake and plant growth, [15], table 1. All over Amazonia, the occurrence of dry periods, through cloud cover reduction, was found to enhance canopy photosynthetic capacity by 25% [16]. But as high levels of irradiance occured in dry season, we may not rule out the possibility that irradiance have a negative effect on tree growth [17].

The effects of rising temperature on the physiology of tropical forest trees are actively debated through the scientific community. Some works suggest that although reductions in photosynthetic rate at temperature above 30°C may occur, these are driven by reductions in stomatal conductance in response to higher leaf-to-air vapour pressure deficits [18], rather than by a direct down regulation of biochemical processes during CO_2_ fixation. Recent studies, however, suggest that tropical tree mortality may increase significantly with increasing night-time temperature while tree growth appears surprisingly sensitive to variations in mean annual night-time temperature of 1–2°C with minimal temperature associated with minimal tree growth [19].

At a daily time step, a high Vapour Pressure Deficit (*VPD*) leads to an inhibition of stomatal conductance in tropical trees [20], [21]. Although gross primary productivity declines with *VPD* for the sparse canopy cover across Amazon, no clear link has been found in densely forested areas [22]. Tropical forest trees don’t have a high sensitivity to *VPD* [23].

Friction velocity (U*) is a climate variable provided by eddy flux data which is correlated with wind speed. A threshold of U*<0.2 m.s^–1^ is generally used to filter eddy covariance data as it is likely that under this value storage and advection can reduce gas fluxes through the boundary layer [24]. CO_2_ can be depleted during the day if mixing is low. In our environment where U* values are rather low, mean of 0.37±0.11 m.s^–1^, and limited by mixing rate with the air above the canopy, as it’s often the case in large tropical forests, high U* leads to high mixing rate and can provide fresh CO_2_ to the depleted within-canopy air and thus increase growth.

In order to predict the potential consequences of currently simulated future climate scenarios [25] on tropical forest dynamics, the challenge is now to rank the potential key climate drivers and to include these drivers into forest dynamic models. With this perspective, large and long-term inventory plots with regular tree census are needed to account for variation in individual growth and in climate patterns [26]. The problem is that most of these long-term studied forests are not adapted to evaluate the climate change impacts because of multi-year census intervals [19]. This impedes our ability to compare data from different years and to study the effect of climate seasonality. In this paper, we used two unique 4-year datasets where bimestrial measurements of tree growth have been recorded on 205 individual trees from 54 neotropical species and where values of climate variables have been daily-averaged (data registered at a half-hourly time step).

This paper has three objectives: (i) to include the climate variables into tree growth models when growth and climate variables are not recorded at the same time step, (ii) to quantify the proportion of observed variance in tree growth that is attributable to climate variations, and (iii) to identify (and rank) the climate variables that most affect tree growth. We hypothesized that in our study site, tree growth is mainly limited by water availability (*REW*), U* and climate variables related to a high evaporative demand (*VPD*, irradiance) rather than temperature.

## Materials and Methods

### Study Site

The study site is located in Paracou, French Guiana (5°18′N, 52°23′W), a lowland tropical rain forest near Sinnamary [27]. This site is part of a private domain owned by the CNES (Centre National d’Etudes Spatiales), and is granted to the CIRAD (Centre de Coopération Internationale en Recherche Agronomique pour le Développement) through an agreement which dedicated the area for forest research activities. The site receives about 2/3 of the annual 3160 mm±161 of precipitation between mid-March and mid-June, and is subject to a 2–3 months dry season around October [14] during which rainfall is less than 50 mm.month^–1^ [28]. The most common soils in Paracou are the shallow ferralitic soils limited in depth by a more or less transformed loamy saprolithe [27]. The site is located approximately 40 m above sea level [27] and is made up of a succession of small hills. The forest is typical of Guianan rainforests [29], [30]. More than 550 woody species attaining 2 cm DBH (Diameter at Breast Height, i.e. 130 cm) have been described at the site, with an estimated 160–180 species of trees >10 cm DBH per hectare. The annual DBH increment averages 0.12±0.01 cm.yr^–1^ and the gain of biommass due to tree growth averages 4.31±0.164 Mg.ha^–1^.yr^–1^ [30], [31]. The dominant families at the site include Leguminoseae, Chrysobalanaceae, Lecythidaceae, Sapotaceae and Burseraceae. No specific permits were required for the following described field studies and this study did not involve endangered or protected species.

### Growth Data

Seasonal changes in trunk circumference were monitored in 205 trees from 54 species using homemade steel dendrometer bands [32]. These trees were located in the footprint area of the Guyaflux tower. Tree growth was censused every c. 40 days from January 2007 to December 2010 (mean = 39 days, sd = 19.8). We defined a categorical variable, *period*, corresponding to each time step between two successive DBH measures. We use diameter growth in the following analysis rather than biomass increement because the distribution of our sampled trees is not representative of the structure of the forest.

### Meteorological Data

**Figure 1:**
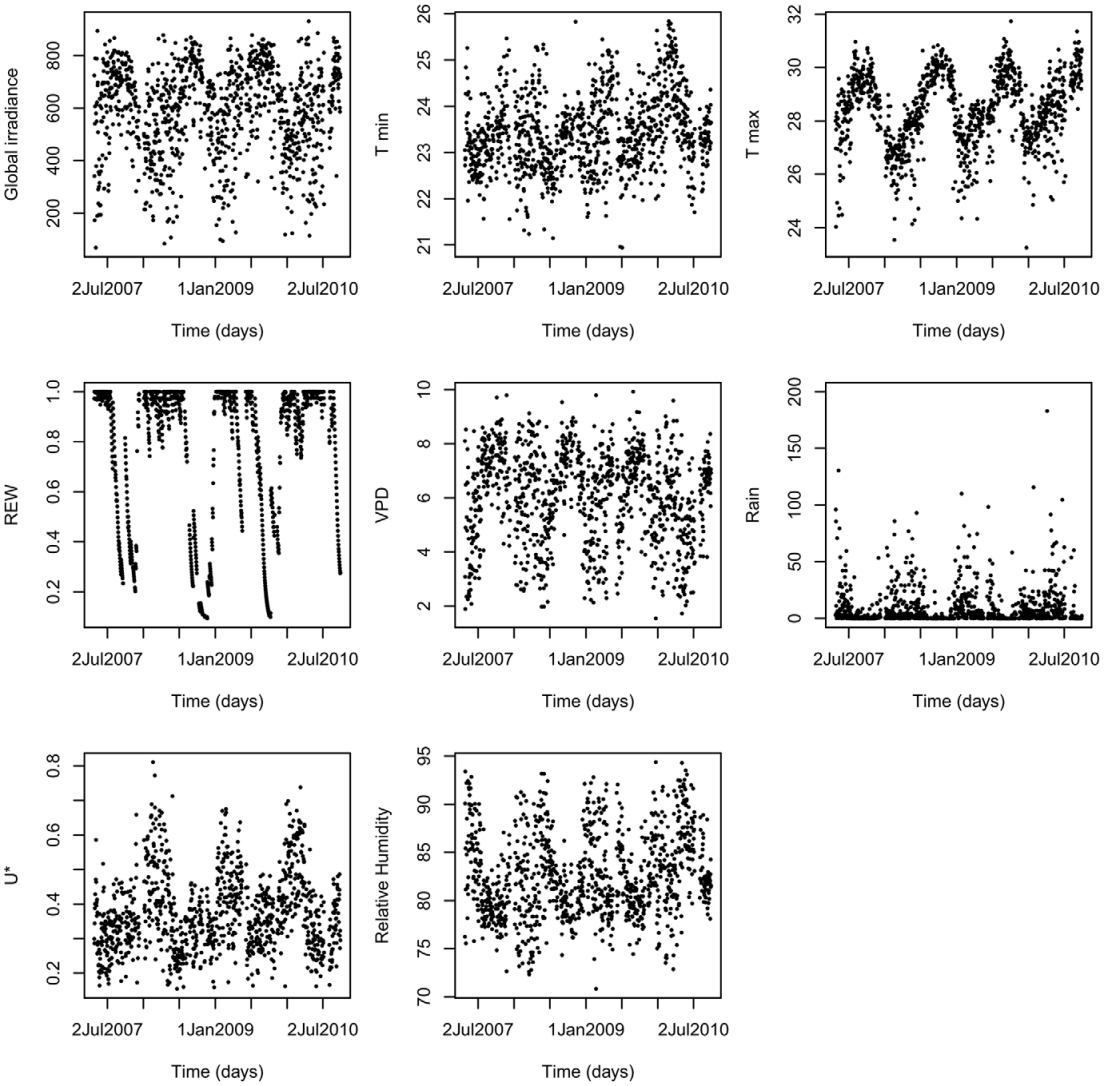
Variation of the climate variables during a 4-year study period in French Guiana. Note that the climate is affected by the north/south movements of the Inter-Tropical Convergence Zone and the site receives nearly two-thirds of its annual 3041 mm of precipitation between mid-March and mid-June, and less than 50 mm per month during the 3-months dry season.

In 2003, a 55 m high self-supporting metallic tower, Guyaflux, was built in the Paracou forest in a natural 100 m^2^ gap [28] in order to measure greenhouse gas exchange between the ecosystem and the atmosphere using the eddy covariance methodology. The top of the tower is about 20 m higher than the overall canopy, and meteorological and eddy flux sensors are mounted 2 m above the tower [28]. A large panel of climate variables was recorded at a half-hourly time-step (details in Table 2). Most climate variables exhibited strong seasonal changes, highlighting the north/south movements of the Inter-Tropical Convergence Zone (Fig. 1).

**Table 2:** Descriptive statistics of the climate variables from 2007 to 2010.

Variable	Daily computation	Description	Mean	SD	Unit
*irradiance*	mean	global irradiance	590.34	170.17	MJ.m^–2^ .d^–1^
*T min*	minimum	temperature minimum	23.43	0.82	celsius degree
*T max*	maximum	temperature maximum	28.37	1.35	celsius degree
*REW*	-	relative extractable water	0.77	0.28	-
*VPD*	mean	vapor pressure deficit	6.05	1.68	kPa
*rainfall*	sum	precipitation	9.03	17.21	mm
*U* ^*^	mean	friction velocity	0.37	0.11	m.s^–1^

The raw data, excepted *REW*, are registered at a half-hourly time-step.

Finally, we used a soil water balance model, developed and validated for tropical forests [14], in order to estimate water availability for trees. The model computes daily water fluxes (tree transpiration, understorey evapotranspiration, rainfall interception and drainage), soil water content at different layers and relative extractable water for trees for the entire soil (*REW*). *REW* is a daily value between 0 and 1: when *REW* = 1, the amount of extractable water by the tree is at its maximum, and when *REW* = 0, no water is available for trees. *Stricto sensu REW* is an environmental variable but, as soil properties and root distribution do not affect the value of *REW* in the Paracou forest [31], *REW* was computed at the forest level and considered as a climate variable in this study.

In a preliminary study, we investigated the association between climate variables through a principle component analysis (PCA) on the normalized climate dataset to describe how the variance of the dataset was structured by the climatic variables and to select representative variables based on correlations between them in order to lower multicollinearity problems in the subsequent analyses.

### Including Climate Variables in Growth Models

We modelled the link between tree growth and climate with a linear regression framework. We first included a factorial variable *tree* in the growth model to take into account the individual behaviours of tree growth. This individual *tree* effect was not further analysed here as the main objective of this study was to analyse the global population pattern rather than the individual tree one; we just took it into account in order to avoid any statistical bias in our results. Next we included the factorial variable *period*, which estimates a model parameter for each period. This reference model, called *m*_0_, was explicitly built to estimate the maximum part of variance that can be imputable to climatic variation, the *period* effect. An arising problem was that periods include different numbers of days, i.e. they did not exactly have the same length. We thus built a seasonal growth model:
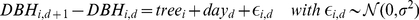
(1)


where *DBH_i_*_,*d*_ is the diameter of tree *i* on the day *d*, *tree_i_* is the individual effect *tree* on daily growth for the tree *i*, *day_d_* is the effect of the day *d* on growth of all the trees and 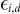 is the error of the model assumed normal. The growth for the tree *i* over the period *j* starting the day *d* and during *nd_j_* days was provided by summing *nd_j_* times equations (1):
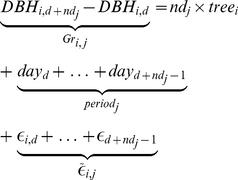
(2)


The equation (2) simplified:
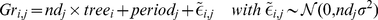
(3)where *Gr_i_*_,*j*_ is the growth of tree *i* over the period *j*, *period_j_* is the effect *period* for period *j*, *nd_j_* is the number of days of period *j* and 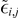 is the error of the model assumed normal. The model (3) was not suited to classical linear regression because the variance 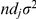 of the error terms changed over the periods *j*. We normalized the equation (3) to reach residual error variance equality, which led to the reference model *m*_0_:
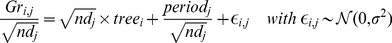
(4)where 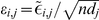 and 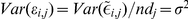.

### Ranking Climate Variables According to their Effect on Tree Growth

We first assessed the ability of the registered climate variables (details in Table 2) to explain the between-periods variance by substituting them to the *period* effect in the reference model *m*_0_. In a first step, we performed univariate analyses for each climate variable by fitting the models *m_varclim_*, for *varclim* = *REW*, *U* ^*^, *T min*, *T max*, *irradiance*, *VPD*, Patm, HR or rainfall, defined by:
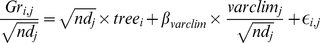
(5)


In a second step, we adopted a multivariate approach and included all the climate variables into a single model, for which we looked for the best model according to the Bayesian Information Criterion (BIC), *m_BIC_* (eq.6).
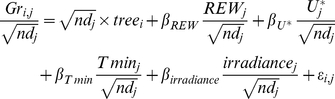
(6)


**Figure 2:**
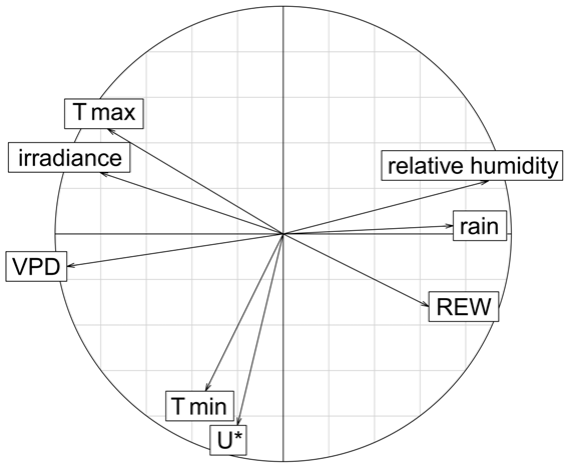
Principal component analysis of the climate variables. Note that the axis 1 and 2 explain respectively 50.8% and 20.6% of the total variation. The third axis explained a further 8.85% of the variance and was linked only to minimal temperature and *REW*.

The model *m_BIC_* was obtained after an exhaustive screening of the candidate models using the package glmulti [33]. We used this package in order (i) to find the best variable linear combination that contains the maximum of information to link growth and climate variables according to the BIC criterion, and (ii) to lower the multicollinearity problem by dropping some climate variables that are highly correlated with each other (Fig. 2). We used BIC, instead of the classically used AIC, to avoid over-parameterization as this criterion is consistent and parsimonious for model selection with respect to large datasets [34].

Finally, we compared the fitted models *m*_0_, *m_varclim_* and *m_BIC_* through their percentage of variance explained and their predictive quality. The later was assessed by computing the root mean square errors of predictions, *RMSEP*:
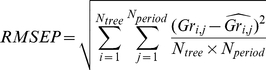
(7)where *Gr_i_*_,*j*_ is the observed values of growth and 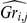 is the model predictions of growth.

All analyses were performed using the R project software (http://www.r-project.org/).

## Results

### Selection of Climate Variables

All climate variables were correlated with at least one other climate variable (Fig. 2). Minimum temperature and U* were intimately linked to each other but moderately with the other climate variables. Maximal temperature was positively correlated with irradiance and negatively with rainfall, relative extractable water and air relative humidity. The correlation circle of the PCA (Fig. 2) highlighted these patterns of correlation, which are mainly due to the strong seasonality of the climate in French Guiana [14], [28], [32]. In order to limit critical correlations between climate variables, we kept in the following analysis the variable for which we have a strong physiological assumption of their effect on tree growth, U*, *REW*, irradiance, *T max* and *VPD*. We also kept rainfall to compare its predictive power with *REW* and minimum temperature due to the previous results of its effect on tree growth [19].

### Model Selection

**Figure 3:**
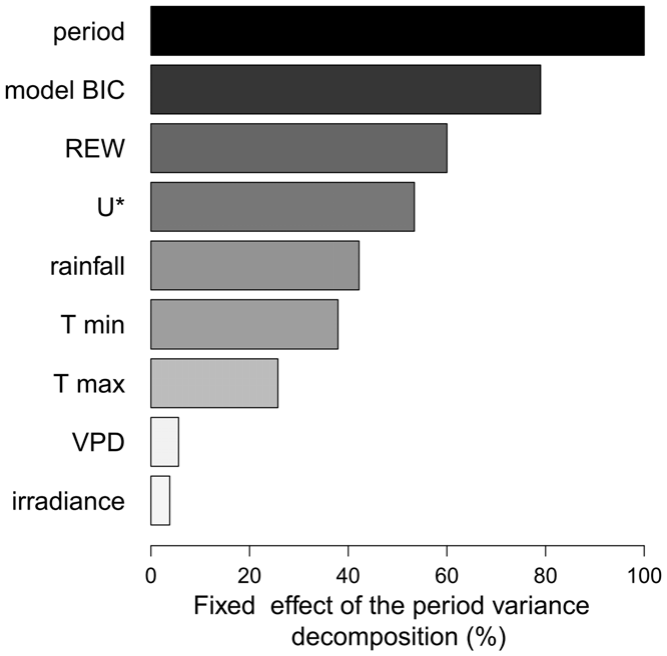
Ranking climate variables according to their effects on diameter growth of 205 trees from 54 neotropical species. The period effect represent 9% of the variance of tree growth. Note that (i) the model BIC catches more than 80% of the period effect and (ii) the water availability (*REW*) alone captures 60% of the period effect, respectively 7.1 and 5.4% of the variance of tree growth.

**Figure 4:**
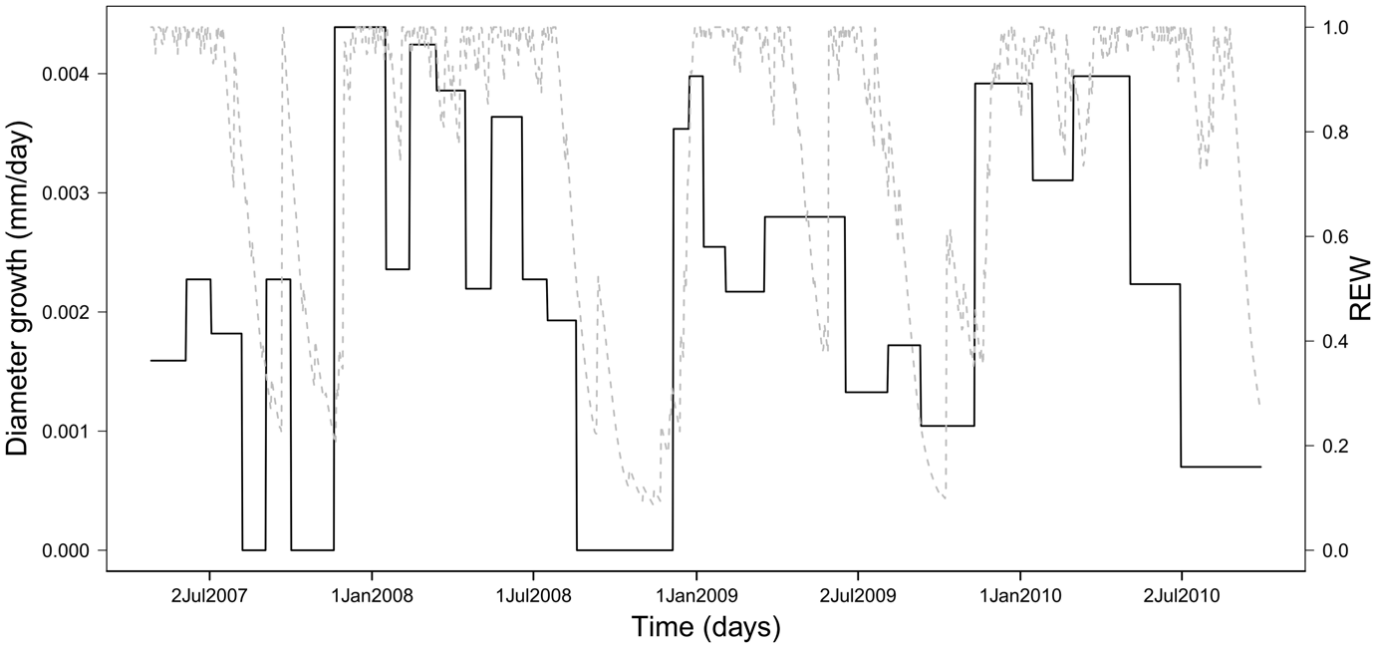
Averaged diameter growth of 205 trees from 54 neotropical species during 4 consecutive years plotted against the evolution of water availability (*REW*, dashed line). Note that the highest increments occur in the first weeks of the wet season, regardless of the intensity of these early rainfall events.

More than 26% of the observed variation in tree growth may be imputable to the individual tree behavior (models *m*_0_, Table 3) while the period effect explained 9% of the tree growth variance. This means that climate variables alone can explain up to 9% of the variance of tree growth. From the univariate analyses (models *m_varclim_*), *REW* explained the largest part of the period effect on tree growth (60%, see Fig. 3). U*, Rainfall or minimum temperature alone explained between 38 and 54% of this period effect. Maximum temperature, *VPD* and irradiance explained less than 26% of the period effect. *REW* was thus, by far, the main predictor of individual tree growth. During the dry season when *REW* decreased below 0.4, the averaged population diameter growths were the smallest within the year and sometimes stopped, Fig. 4. At the beginning of the rainy season, *REW* and tree growth quickly increased simultaneously. However, at the end of the dry season, tree growth and *REW* did not exhibit the same pattern, i.e. the averaged individual tree growth began to decline before the *REW* itself diminished.

**Table 3:** Variance decomposition of the univariate analysis.

model	components	Df	Sum Sq	Mean Sq	% of variance	F value	P value
M_0_							
	*tree*	204	2.9130	0.0142795	26.1972	11.472	<0.001
	*period*	35	0.9970	0.0284864	8.966375	22.885	<0.001
	*residuals*	5792	7.2095	0.0012447	64.83643		
M*_REW_*							
	*tree*	204	2.9130	0.01428	26.1972	10.934	<0.001
	*REW*	1	0.5983	0.59833	5.380854	458.170	<0.001
	*residuals*	5826	7.6082	0.00131	68.42195		
M*_rain_*							
	*tree*	204	2.9130	0.01428	26.1972	10.685	<0.001
	*rain*	1	0.4206	0.42064	3.782924	314.758	<0.001
	*residuals*	5826	7.7859	0.00134	70.01988		
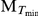							
	*tree*	204	2.9130	0.01428	26.1972	10.627	<0.001
	*T min*	1	0.3781	0.37809	3.400231	281.378	<0.001
	*residuals*	5826	7.8285	0.00134	70.40257		
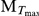							
	*tree*	204	2.9130	0.014279	26.1972	10.464	<0.001
	*T max*	1	0.2566	0.256635	2.307954	188.071	<0.001
	*residuals*	5826	7.9499	0.001365	71.49485		
M*_VPD_*							
	*tree*	204	2.9130	0.014279	26.1972	10.207	<0.001
	*VPD*	1	0.0559	0.055886	0.5025887	39.946	<0.001
	*residuals*	5826	8.1507	0.001399	73.30021		
M*_rg_*							
	*tree*	204	2.9130	0.014279	26.1972	10.184	<0.001
	*irradiance*	1	0.0380	0.038042	0.3421205	27.133	<0.001
	*residuals*	5826	8.1685	0.001402	73.46068		
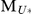							
	*tree*	204	2.9130	0.014279	26.1972	10.184	<0.001
	U*	1	0.5325	0.53248	4.7886	404.247	<0.001
	*residuals*	5826	7.6741	0.00132	69.01414		
M*_BIC_*							
	*tree*	204	2.9130	0.01428	26.1972	11.210	<0.001
	*REW*	1	0.5983	0.59833	5.380854	469.699	<0.001
	*T min*	1	0.0154	0.01781	0.1387580	12.112	<0.001
	*irradiance*	1	0.1739	0.17388	1.563707	136.497	<0.001
	*Residuals*	5823	7.4189	0.00127	66.71948		

The selection procedure using the BIC criterion kept 3 climate variables in the final multivariate model *m_BIC_*: *REW*, minimum temperature and irradiance, Table 4. The proportion of variance imputable to each climate variable as well as the values of the model parameters were hard to interpret in the *m_BIC_* model because climate variables were highly correlated. However, from the univariate analyses, we determined that all climate variables have positive parameters indicating a positive effects on tree growth (Table 4). All in all, the variance of the period effect on individual tree growth is well-explained by the final model *m_BIC_*, with 79.0% of variance of the period effect, i.e. 7.1% of the tree growth variance, explained by the combination of these three climate factors (Fig. 3).

**Table 4:** Model parameters, standard errors and t values of the univariate (*m_varclim_*) and final multivariate (*m_BIC_*) analyses.

model	climate variable	estimate	Std. Error	t value	P value
*m_varclim_*					
	*REW*	4.658×10^−3^	2.176×10^−4^	21.405	<0.001
	*rainfall*	2.015×10^−4^	1.136×10^−5^	17.741	<0.001
	*T min*	2.209×10^−4^	1.317×10^−5^	16.774	<0.001
	*T max*	1.532×10^−4^	1.117×10^−5^	13.714	<0.001
	*VPD*	3.028×10^−4^	4.791×10^−5^	6.320	<0.001
	*irradiance*	2.255×10^−6^	4.330×10^−7^	5.209	<0.001
	U*	1.241×10^−2^	6.175×10^−4^	20.106	<0.001
*m_BIC_*					
	*REW*	1.628×10^−3^	3.542×10^−4^	4.596	<0.001
	*T min*	4.471×10^−4^	3.739×10^−5^	11.959	<0.001
	*irradiance*	−1.107×10^−5^	9.474×10^−7^	−11.683	<0.001

Models *m_varclim_* were separately fit for each climate variable.

### Model Predictions

**Figure 5:**
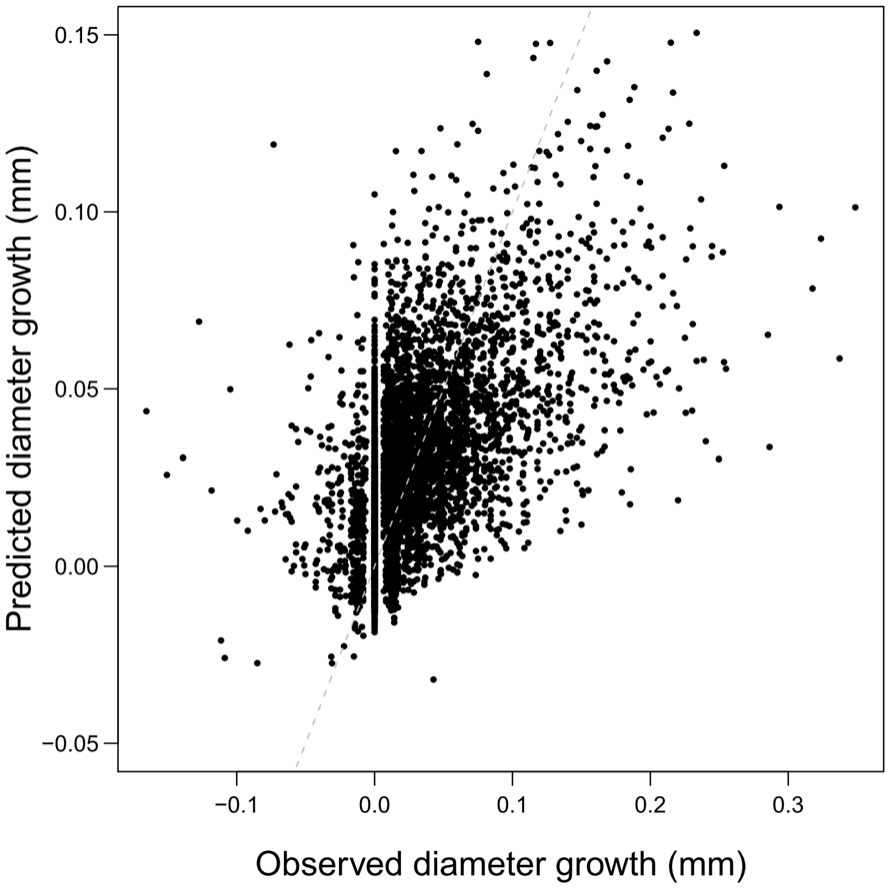
Predicted versus observed diameter growth under the model m_BIC_. The white dashed line is the identity line *y* = *x*. Note that the model overestimated the individual growth under 0.05 mm.d^−1^ and underestimated the growth above 0.1 mm.d^−1^.

The model *m_BIC_* did not completely succeed in accurately predicting the seasonal growth. Indeed, the obtained RMSEP was slightly above the mean value of growth (mean growth = 0.026 mm.d^−1^, RMSEP = 0.035 mm.d^−1^). In general, the model overestimated the individual growth under 0.05 mm.d^−1^ and underestimated the growth above 0.1 mm.d^−1^ (Fig. 5).

## Discussion

In this study, we showed that 9% of the observed variation in individual tree growth was attributable to fine-scale climate variations and demonstrated that water availability was the main driver of tree growth in a neotropical forest. The individual behaviour of each tree explained 26% of growth variation and a substantial fraction of variation in growth remained unexplained with our model. Tree growth is obviously influenced by several additional environmental variables, such as competition for light and nutrients [35]–[37]. Their effects were included in the model through the individual effect *tree_i_* and we assumed that they were constant over the study period. We also did not consider the ontogenetic trajectory that depends on complex environmental changes that may have occurred during the 4-year census period [38]. The remaining unexplained 65% of tree growth variance could be linked (i) to complex changes in environnemental conditions during the experiment, such as change in light availability created by a new forest gap, or (ii) to complex biological properties, such as the inherent rhythm of leaves and flowers phenology or changes in ontogenetic growth trajectory. In this study, we made the strong assumption that each growth measurement was independent of others. For instance, a single heavy rain in dry season does not have the same effect than a single heavy rain in wet season. Further research should improve these components of the model to take better account of seasonal change of tree growth. For example, future predictions of the effects of precipitation variability on carbon assimilation and on tree growth could be improved by the use of tree hydrodynamic models that mechanistically relate tree transpiration and stomatal conductance to soil moisture, through resolving water stresses in the tree system [39], [40].

### Soil Water Availability

Soil water availability strongly impacts productivity as directly observed in seasonal tropical forests [41], [42] and as deduced from experimental forest droughts [12], [43]. In our study where tree growth was linked to soil water availability at a seasonal time step, we were able to go further than earlier studies performed at an annual scale [10], [44]. Indeed, our methodological approach allowed us to rank the effects of the different climate variables tested here on tree growth. We showed that low levels of *REW*, rather than lack of rainfall, are the key driver of the decrease, or even the stop, of diameter increment. This result thus points to the main influence of soil water availability on the biological processes (i.e. cell division in cambial tissues) associated with secondary growth of tropical rainforest species. However, Stahl *et al.* [32] even highlighted a shrinkage of the circumference of some trees during dry seasons at the same site and concluded that seasonal variations in tree circumference partly reflect variation in trunk biophysical properties. Deciphering the relative importance of stem shrinkage and/or decrease in diameter growth in dry seasons was beyond the scope of this work and supplementary in situ experiments are needed. Nevertheless, the Paracou forest experienced strong dry seasons during the study period with even several months with precipitation <50 mm.month^−1^ (0–4 month.year^−1^). During these events, the amount of rainfall was always below the potential evapotranspiration, which never falls below 100 mm.month^−1^ in Paracou [45]. This water limitation may solely explain the slowdown of girth increment, as reported in many seasonal tropical forests [42]. However, even under strong water limitation, most trees seem to be able to maintain their baseline functioning [46] and the decrease in gross ecosystem productivity under severe dry conditions did not exceed 20% of wet season values at the Paracou site [28]. The apparent discrepancy between high ecosystem-level gross productivity and low community secondary growth at our site can be explained by a large proportion of the photosynthate products stocked into reserve pools under soil drought conditions [47]. The strongest growth increments occurred (see Fig. 5) during the early wet season. The same pattern has already been observed at La Selva leading Clark et al. [19] to conclude a strong link between growth and rainfall. However, a direct effect of rain on biological processes leading to secondary growth is rather hypothetical, and other processes are involved to explain this relationship. In a tropical forest of Ethiopia with a strong seasonality, high-resolution electronic dendrometers have been combined to wood anatomy investigation to describe cambial growth dynamics [48]. These authors have observed that lack of water availability during the long dry season induced cambial dormancy. Furthermore, after the onset of the rainy season, (i) bark swelling started quite synchronously among trees, (ii) bark swelling was maximum after a few rainy days and (iii) evergreen trees were able to quickly initiate wood formation. Namely, we still do not know whether this increment is due to cambial activity, sapwood or bark swelling or, more probably, a combination of these [32]. A flush of nutrient availability at the start of the rain season may also explain this swift diameter increment as the first rainfall events make available a large pool of nutrients accumulated during the dry season [49].

### Temperature

Investigating the effects of temperature on the physiology of tropical forest trees [18], [50] is, today, of primary importance, given increases expected over the next century [25], [51]. Some authors suggest that tropical trees are, more than others, sensitive to temperature increases because (i) they live at or close to the highest annual average temperatures on Earth and (ii) tropical species naturally encounter limited variation in temperature (<4°C over 20° of latitude) [52]. In French Guiana, the increase in average temperature follows the general trend of Amazonia, 0.25±0.05°C per decade [1], [53]. This increase in average temperature is mainly driven, in French Guiana, by the minimum daily temperatures over the last 50 years (unpublished data). We found that temperature variations were of secondary importance for tree growth at a seasonal time step. Nevertheless, minimal temperature was slightly positively correlated with tree growth (Fig. 3), whereas maximal temperature had no effect. The interpretation of this significant correlation is rather biologically difficult, as minimal daily temperature still remain rather high at our site (never less than 21°C) and seasonal variations in these temperatures remain rather low. At La Selva [19], annual growth was found to be sensitive to variations in mean annual night-time temperature of 1–2°C. However, we argue that the climate at La Selva is near aseasonal with no strong dry periods and tree diameter increment never really stops. Thus, the observed relationship at La Selva may have arisen because night-time temperature is a proxy of drought events, as the census with the strongest dry season was the census with the highest mean annual night-time temperature [19], [54].

### Irradiance

Surprisingly, amongst the climate variables that were significantly correlated with seasonal growth variation, irradiance had the smallest effect on tree growth (Fig. 3). Gross primary production is limited by irradiance in the Paracou forest, but the critical level where irradiance becomes limiting is rarely attained [28]. In the final model selected by BIC, irradiance had a surprising negative effect on growth. In fact, the model used irradiance to lower tree growth during the strongest dry seasons. These extreme slowdowns of tree growth could be linked to the leaf fall phenology mediated by high irradiance events as previously observed in Tapajos forest [46], [55]. Irradiance has been previously reported to be the main determinant of leaf fall timing in aseasonal [56] as well as in seasonal rainforests [57], [58]. However, in Paracou, litter production remains high all over the year [28], [59] and a peak is observed around September [28], [60], i.e. when irradiance is the highest. This could lead us to conclude that the massive loss of leaves in September led to a decrease in the whole-ecosystem photosynthetic capacity, in turn driving the growth slowdown. However, enhanced vegetation index (EVI, a proxy of chlorophyll activity) is also highest during this period due to the establishment of newly formed leaves [60]. In this context, the negative link between irradiance and tree growth should be cautiously interpreted and we suggest that future work should be carried out to test this phenology effect on tree growth.

### Conclusions

Globally, current IPCC scenarios predict an intensification of the dry period for the Guiana shield [25] during the XXI*^st^* century. Amongst climate variables, our results highlight the predominant role of water availability in determining tree growth. If rainfall reduction was confirmed in the future, it may be expected that tree growth will be affected. 91% of the variance in stem increment unresolved may be due to neotropical trees acclimation to quick changing environmental conditions at Paracou or more likely to the limitations of our modeling approach that does not account for biological lags. While photosynthesis or gross primary production often adjusts immediately to environmental conditions, structural growth may be correlated with environmental conditions occurring weeks to years earlier [60]–[65]. Indeed, dry periods lead to an effective decrease in stem diameter growth but their actual intensity hardly affects the global annual tree growth that appears more dependent on the increment at the onset of the rain seasons than on the duration of the dry seasons. In other words, six or nine months of rain season seems to be equivalent as the highest increments occur in the first weeks of the wet season, regardless of the intensity of these early rainfall events (Fig. 4). In this context, it seems necessary to explicitly include the effect of water stress on tree growth in forest simulators to test, *in silico*, the impact of different climate scenarios on the future dynamics of the rainforest.
